# Genetic predisposition to immune dysregulation and extracellular matrix remodeling in cardiac arrhythmia reveals potential mediation by *SPP1*+ macrophages

**DOI:** 10.3389/fcell.2025.1611663

**Published:** 2025-08-18

**Authors:** Jie-Yuan Jin, Shuai Guo, Yao Deng, Ya-Qin Chen, Chen Liang, Yu-Jie Jiang, Wang Zhao, Rong Xiang

**Affiliations:** ^1^ School of Medicine, Shaoxing University, Shaoxing, China; ^2^ School of Life Sciences, Central South University, Changsha, China; ^3^ Department of Cardiovascular Surgery, Xiangya Hospital, Central South University, Changsha, China; ^4^ Department of Cardiovascular Medicine, the Second Xiangya Hospital, Central South University, Changsha, China; ^5^ Center for Medical Genetics, Jiangmen Maternal & Child Healthcare Hospital, Jiangmen, China

**Keywords:** cardiac arrhythmias, whole-exome sequencing (WES), single-cell transcriptomics, SPP1+ macrophage, immune dysregulation, extracellular matrix remodeling, cardiomyopathy, myocardial fibrosis

## Abstract

**Introduction:**

Cardiac arrhythmia frequently co-presents with structural abnormalities such as cardiomyopathy and myocardial fibrosis, creating a bidirectional relationship where electrical disturbances and structural remodeling exacerbate each other. Current genetic studies focus on ion channel variants, which explain part of the etiology. Molecular mechanisms underlying arrhythmias pathogenesis and its progression warrant further investigation.

**Methods:**

We performed whole-exome sequencing on 50 arrhythmia patients (21 females, 29 males), predominantly with early-onset disease (94% ≤ 35 years). We focused on exonic deleterious mutations that are rare in healthy populations. The identified recurrently mutated (*r.m.*) genes were analyzed using protein-protein interaction networks and gene ontology enrichment for functional modules. These genomic insights were integrated with single-cell data (7 arrhythmias, 5 controls) to examine cell-type-specific gene expression changes, with particular focus on *SPP1*+ macrophage states.

**Results:**

We identified 132 *r.m.* genes present in ≥30% of patients in our cohort, with significant functional module enrichment in immune regulation, tissue homeostasis, extracellular matrix, and vesicle transport pathways. Single-cell analysis of 37,675 cells revealed conserved transcriptional signatures across cell types, characterized by enhanced cytokine responses and pro-fibrogenic programs. We discovered genetic determinants potentially underlying *SPP1*+ macrophage activation in arrhythmic hearts—a known mediator implicated in both inflammatory processes and fibrotic remodeling. Age-specific associations included *ADAMTS7* mutations in very early-onset cases (≤20years; OR = 9.71 [2.38–47.74], *P-value* <0.001), while gender-specific variants included *SLC9B1* (*P-value* = 0.017) exclusively in females. Additionally, *OTOA* mutations were associated with both relatively late onset (>20years; OR = 0.17 [0.04–0.68], *P-value* = 0.009) and female predominance (OR = 3.41 [0.92–13.58], *P-value* = 0.045).

**Conclusion:**

Our exploratory analysis reveals how genetic variants may predispose arrhythmia patients to inflammatory and fibrotic processes. These findings may help guide future research into the molecular mechanisms underlying arrhythmia progression to structural heart disease and identify candidate pathways for therapeutic investigation.

## 1 Introduction

Cardiac arrhythmia is one of the most prevalent cardiovascular diseases, referring a spectrum of disorders characterized by irregularities in the heart’s rhythm, frequency, origin of impulses, conduction velocity, and sequence of excitation (Schwartz et al., 2020). It contributes significantly to the global burden of cardiovascular morbidity and mortality (Khurshid et al., 2018). The rise in arrhythmia incidence demands improved clinical management (Boriani et al., 2021). However, the complexity of arrhythmia poses significant challenges in developing effective treatments (Boersma et al., 2023). Clinically, arrhythmias frequently co-present with structural heart abnormalities such as cardiomyopathy (Shoureshi et al., 2024) and myocardial fibrosis (Sohns and Marrouche, 2020), and may either result from or contribute to these conditions (Boersma et al., 2023; Keating and Sanguinetti, 2001; Lukas Laws et al., 2022). This bidirectional relationship creates a potential vicious cycle (Piek et al., 2019) where electrical disturbances promote structural remodeling, which in turn exacerbates arrhythmogenicity. Therefore, the molecular mechanisms governing the progression from isolated arrhythmias to structural heart disease warranted further investigation.

The etiology of cardiac arrhythmia involves genetic predispositions, environmental factors, and structural heart diseases (Boersma et al., 2023; Keating and Sanguinetti, 2001; Lukas Laws et al., 2022) (e.g., cardiomyopathy and cardiac fibrosis). Genetic variations are key contributors to arrhythmias (Schwartz et al., 2020), with numerous risk genes primarily involved in critical cardiac functions such as ion channel activity, ion pump regulation, adrenergic signaling, intercellular communication, and intracellular signal transduction (Lukas Laws et al., 2022; Roselli et al., 2020; Marian et al., 2020). The variants in ion channel genes, including sodium channel genes (*SCN5A*, *SCN1B*, *SCN2B*), potassium channel genes (*KCNK1*, *KCNE1*, *KCNE2*), and calcium channel genes (*CACNA1D*, *CASQ1*, *RYR2*), are well documented. Additionally, genes related to the Na+/K+ pump (e.g., *ATP1A1*), adrenergic receptors (e.g., *ADRA*, *ADRB1*), and structural proteins (e.g., *FKBP1B*, *GJA1*, *ANK2*) contribute to the arrhythmic phenotype. These cannot fully explain why some patients develop progressive structural disease while others maintain normal cardiac morphology. The conventional focus on ion channel abnormalities accounts for electrical disturbances but fails to explain fibrotic remodeling and myocardial dysfunction in chronic arrhythmia patients, suggesting additional genetic and molecular factors predispose certain individuals to disease progression.

Recent studies have identified nontraditional factors in arrhythmia pathogenesis, including inflammatory processes (Grune et al., 2021; Hulsmans et al., 2023) and gut microbial-host crosstalk (Fan et al., 2023; Witkowski et al., 2020). Single-cell studies (Hulsmans et al., 2023; Selewa et al., 2023) reveal significant alterations in inflammatory pathways in cardiac arrhythmias, particularly noting an expansion of inflammatory monocytes and secreted phosphoprotein 1 (*SPP1+*) macrophages. Experimental models (Hulsmans et al., 2023), the *CCR2*-null HOMER mice, have demonstrated that inhibiting monocyte migration can reduce arrhythmic events, underscoring the therapeutic value of targeting inflammatory pathways. Notably, SPP1 protein (also known as osteopontin) functions as a multifaceted signaling molecule involved in both immune modulation and extracellular matrix remodeling, linking inflammation to fibrogenesis. However, the genetic determinants driving activated *CCR2*/*SPP1* axis and how these processes contribute to structural remodeling remain unknown. The gap between genetic variations and their influence on cardiac microenvironment transcriptional landscapes significantly hampers our understanding of arrhythmogenesis and its progression.

Our study bridges this critical knowledge gap by exploring the crosstalk between genetic predispositions and cellular alterations in arrhythmias. Analysis of our patient cohort reveals 132 recurrently mutated (*r.m.*) genes with significant enrichment in four key pathways: immune regulation, tissue homeostasis, ECM/cytoskeleton components, and transport vesicle function. Through scRNA-seq analysis, we observe both pro-inflammatory and pro-fibrogenic transcriptional signatures across multiple cell types. This study uncovers potential genetic drivers of pathological events in cardiac arrhythmias, particularly the *SPP1+* macrophage-mediated inflammation and fibrosis. By illuminating the molecular pathways underpinning both electrical disturbances and structural remodeling, our findings may guide the development of targeted therapeutic strategies addressing the full spectrum of arrhythmia pathology.

## 2 Materials and methods

### 2.1 Subjects design and population

The cohort for this study comprised 50 patients diagnosed with arrhythmia at Xiangya Hospital, Central South University. These patients were enrolled for WES based on the attending clinician’s judgment, typically involving early-onset arrhythmia, atypical clinical presentation, or suspected genetic etiology. All participants were of Han Chinese descent and were recruited in a consecutive manner. Ethical approval for this research was obtained from the Review Board of Xiangya Hospital of Central South University (approval number: 202103427 for human specimens). Informed consent was obtained from all subjects prior to their inclusion in the study. Comprehensive clinical data, encompassing medical and family histories, were collected and documented for each participant, detailed in Supplementary Table S1.

### 2.2 DNA extraction and sequencing experiments

Peripheral blood samples were collected from all study participants. Genomic DNA was extracted from collected samples using the DNeasy Blood and Tissue Kit (Qiagen, Valencia, CA, USA), following the manufacturer’s protocol. WES was conducted at the Beijing Genomics Institute (Beijing, China). The exonic regions were enriched using Agilent SureSelect Human All Exon V6 kits. Subsequently, high-throughput sequencing was performed on an Illumina HiSeq2000 platform, adhering to standard protocols.

### 2.3 Whole exome data processing

Raw sequencing data were evaluated for quality using FastQC (Babraham Bioinformatics, 2019) (v0.11.9). Adapter sequences and low-quality bases were trimmed using TrimGalore (Babraham Bioinformatics group, 2024) (v0.6.6). The processed reads were aligned to the human reference genome (GRCh38/hg38) using the Burrows-Wheeler Aligner (Li and Durbin, 2010) (BWA-MEM, v0.7.17). After alignment, SAMtools (Li et al., 2009) (v1.11) was used to sort the reads and remove duplicates. Base quality score recalibration (BQSR) and local realignment around Indels were conducted using the Genome Analysis Toolkit (GATK, v4.1.9.0), following the best-practice guidelines. Variant calling was performed with GATK HaplotypeCaller, generating single nucleotide variants (SNVs) and small insertions/deletions (Indels) in the variant call format (VCF). Joint genotyping was applied across all samples, and a variant quality score recalibration (VQSR) approach was employed to filter potential false-positive calls. Only high-confidence variants passing the default threshold (FILTER = PASS) were retained. The resulting VCF files were normalized and filtered with following criteria: allele balance of 0.2–0.8 for heterozygous genotypes, genotype quality ≥20, and read depth (DP) ≥10 (≥5 for sex chromosomes). Filtered variants from all individuals were merged for generating the initial arrhythmia genetic dataset for subsequent analyses. We achieved a median sequencing depth of 110x for targeted exonic regions and a minimum coverage of 20x for 99% of the targeted sequences. Our data provided a high level of confidence for identifying variants with potential functional significance, setting the stage for a comprehensive analysis of the potential genetic architecture underlying cardiac arrhythmia in this cohort.

### 2.4 Mutation annotation and prioritizing

Our study focused on rare, deleterious exonic variants. A multi-step selection strategy was applied (Guo et al., 2023; Lin et al., 2025) (Supplementary Figure S1). To distinguish between potentially pathogenic variants and common polymorphisms, we utilized population frequency data from multiple databases: the 1000 Genomes Project (2,504 individuals from 26 populations), gnomAD (v2.1.1, >140,000 individuals), the ExAC database (>60,000 individuals), and our in-house cohort of 100 healthy controls. Variant calling was refined by excluding variants with population variant allele frequencies (VAF) ≥0.01 in any of these databases, as these frequently observed variants are likely to be benign polymorphisms. Variants with population frequencies not available (NA) were assigned a frequency of zero. Non-exonic variants, including intergenic, intronic, and synonymous SNPs, were also filtered out. Focusing on exonic single nucleotide variations (SNVs) and insertions/deletions (Indels) with the potential to alter amino acid sequences and protein structures, we included 42,179 SNVs and 1,945 frameshift Indels in the study. To assess pathogenicity, variants were subjected to a rigorous computational prediction process using four widely recognized algorithms: SIFT (Ng and Henikoff, 2003), MutationTaster (Steinhaus et al., 2021), Polyphen2 (Adzhubei et al., 2013) (in both HumDiv and HumVar modes), and CADD (Rentzsch et al., 2019). The cutoff values for variant pathogenicity were established at 0.05 for SIFT, 0.435 for Polyphen2-HumDiv, 0.445 for Polyphen2-HumVar, and 5 for CADD. Variants predicted benign by at least three of the five tools were categorized as consensus benign, leading to the exclusion of 18,718 variants from downstream analysis. Our procedure yielded 25,406 rare deleterious SNVs and Indels for downstream analysis. The majority were missense variants (non-synonymous variants, n = 21,910; 86.24%), followed by a smaller proportion of nonsense variants (stop gain variants, n = 1,512; 5.95%), stop loss (n = 33; 0.13%), splicing region variants (n = 6; 0.02%), and frameshift indels (n = 1,945; 7.66%). Variants were also annotated with OMIM (Hamosh et al., 2005) and ClinVar (Landrum et al., 2018) databases following their standard tutorial to assess known disease associations and clinical significance.

### 2.5 Protein-protein interaction (PPI) and gene ontology (GO) analysis

Protein-Protein Interaction analysis was conducted using the STRING (Szklarczyk et al., 2023) database following their tutorial (https://string-db.org/). We used the 132 *r.m.* genes plus genes of interest (*SPP1*, *CCR2,* and *CD44*) as input to assess the direct and indirect associations between proteins. The analysis parameters were set to include all active interaction sources such as text mining, experiments, databases, co-expression, neighborhood, gene fusion, and co-occurrence. The confidence score cutoff was established at a high threshold (0.7) to ensure the specificity of interactions. For visualization purposes, the disconnected nodes were hidden from the final network. We also used k-means clustering method imbedded in STRING to cluster interconnected protein nodes. The PPI network visualization highlighted the potential functional connectivity between the identified proteins and their possible collective role in the pathogenesis of cardiac arrhythmias.

GO analysis was carried out using the *ClusterProfiler* (Xu et al., 2024) package in R to categorize the identified *r.m.* genes into biological processes and cellular components. We performed the GO analysis following the recommended tutorial (https://learn.gencore.bio.nyu.edu/rna-seq-analysis/gene-set-enrichment-analysis/). *P*-values were adjusted by Benjamini–Hochberg procedure (Benjamini and Hochberg, 1995) with a cutoff of <0.05 considered significant. The results were visualized in bar plots displaying the -log10 adjusted *P-value*s, which highlighted the most significantly enriched GO terms in the context of biological processes and cellular components.

### 2.6 Single-cell transcriptomics data analysis

Single-cell RNA sequencing data were obtained from the GEO database under accession number GSE224959 (Hulsmans et al., 2023). Analysis of the scRNA-seq data was conducted using the R package Seurat (Hao et al., 2021) (version 5.0.0), following the QC steps outlined in our previous work. Standard procedures, including data normalization, variable gene identification, scaling, and principal component analysis (PCA), were performed. Sample-wise batch effects were removed using the Seurat integration method (CCA mod). The annotation of cell populations within the normal and arrhythmia groups was performed with the canonical cell type makers and can be found in Supplementary Figure S4. Differential expression (DE) analysis between normal and arrhythmia groups was carried out for each identified cell type using the *FindAllMarkers* function within Seurat. This is to identify the cell-type-specific conditional DE genes. Subsequently, GO enrichment analysis was conducted for the upregulated and downregulated genes in each cell type, utilizing *ClusterProfiler* as described in the previous section. Cell-cell communication analysis was performed using the CellChat (Jin et al., 2025) R package (v1.6.1) following the standard tutorial.

### 2.7 Statistical analysis

All statistical analyses were performed using R (v4.0.0). Continuous variables were compared using *Student’s* t*-test*. Clinical association analysis was conducted to identify correlations between genetic variants and clinical characteristics (e.g., disease onset, gender, and cardiomyopathy presence). The associations were assessed using Fisher’s exact test in R with function *fisher.test*. Variants were plotted against their respective chromosomal positions in a Manhattan plot, generated using the *qqman* package in R. The threshold for significance was set at a *P-value* of 0.05. A forest plot was created to visualize the odds ratios (ORs) and their confidence intervals (CIs) for variants associated with specific clinical features using the *forestplot* package in R. We note that in some cases where mutations were absent in one subgroup, OR calculations resulted in extreme values (either 0 or +), which posed challenges for log_2_ transformation in forest plot visualization. To mitigate this, OR values were capped at 0.01 for lower-bound cases and 100 for upper-bound infinite values. This capping strategy is only for data visualization purposes. Mutational hits analysis was conducted to map the distribution of mutational hotspots along the protein sequences of genes identified to harbor variants of interest. Protein length information was retrieved using the *biomaRt* package in R. Mutations were then positioned along the protein sequences according to their amino acid change coordinates. Each variant was plotted to reflect its position within the protein sequence and its recurrence within our patient cohort. The results of this analysis were summarized in a series of lollipop plots, with each lollipop representing a mutation and its frequency. *P*-values were adjusted using the Benjamini–Hochberg (Benjamini and Hochberg, 1995) false discovery rate (FDR) correction method.

## 3 Results

### 3.1 Patient characteristics and WES experiments summary

**FIGURE 1:**
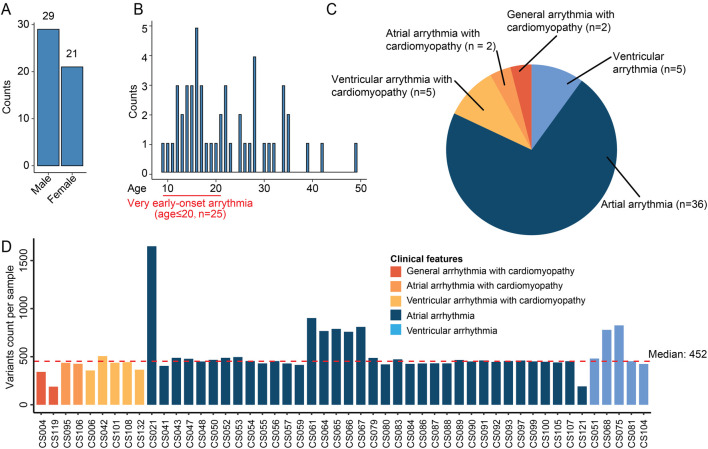
Overview of the cardiac arrhythmia WES dataset. **(A)** Bar plot showing the number of cases per gender. **(B)** Distribution of onset age in the patient cohort. The histogram displays the patients count per age. A highlighted section at the beginning of the age axis denotes patients with very early-onset arrhythmia (age ≤20 years, n = 25). **(C)** Pie chart summarizing the proportion of patients with different types of cardiac arrhythmias. **(D)** Bar plot showing variants count per sample. Each bar corresponds to a sample and color indicates different clinical features. The red dashed line is the median variants count across samples.

In our study, 50 unrelated Chinese individuals diagnosed with cardiac arrhythmia were analyzed (Table 1). The cohort included 21 females and 29 males, with ages ranging from 9 to 49y (mean: 22y) (Figures 1A,B). Notably, 94% of patients presented with early-onset arrhythmias (age ≤35y). This predominance of young patients minimizes environmental confounding factors and emphasizes genetic determinants. Half of our cohort (n = 25) exhibited very early-onset disease (age ≤20years). As arrhythmias are frequently associated with cardiomyopathy, we analyzed these clinical relationships in our cohort (Figure 1C). The majority (72%) presented with isolated atrial arrhythmia, while ventricular arrhythmia showed a striking association with structural heart disease, with cardiomyopathy present in 50% of ventricular cases compared to merely 5.3% of atrial cases. This pattern aligns with the established clinical observation that ventricular arrhythmias typically present with more severe clinical manifestations and stronger connections to underlying structural abnormalities. Detailed clinical information is in Supplementary Table S1.

**TABLE 1:** Clinical features of arrhythmia cohort (n = 50).

Characteristics	
Gender	
Female	21 (42%)
Male	29 (58%)
Age (years)	22.16 ± 9.21
Category	
Atrial arrhythmia	38 (76%)
Ventricular arrhythmia	10 (20%)
General	2 (4%)
Cardiomyopathy	
Positive	9 (18%)

We performed WES experiments on peripheral blood samples per patient. WES data was preprocessed and filtered following our established procedures (Guo et al., 2023; Lin et al., 2025) (Supplementary Figure S1), with a focus on exonic rare deleterious variants. After quality controls, the median variant count per sample was 452, with most samples showing consistent mutational burden (Figure 1C). One outlier, CS021, harbored 1,649 variants. We observed no significant correlation between total mutation burden and patient demographics (age, gender) or clinical disease categories, suggesting that qualitative rather than quantitative genetic differences may underlie the pathophysiological mechanisms in this cohort.

### 3.2 Landscape of recurrently mutated genes

**FIGURE 2:**
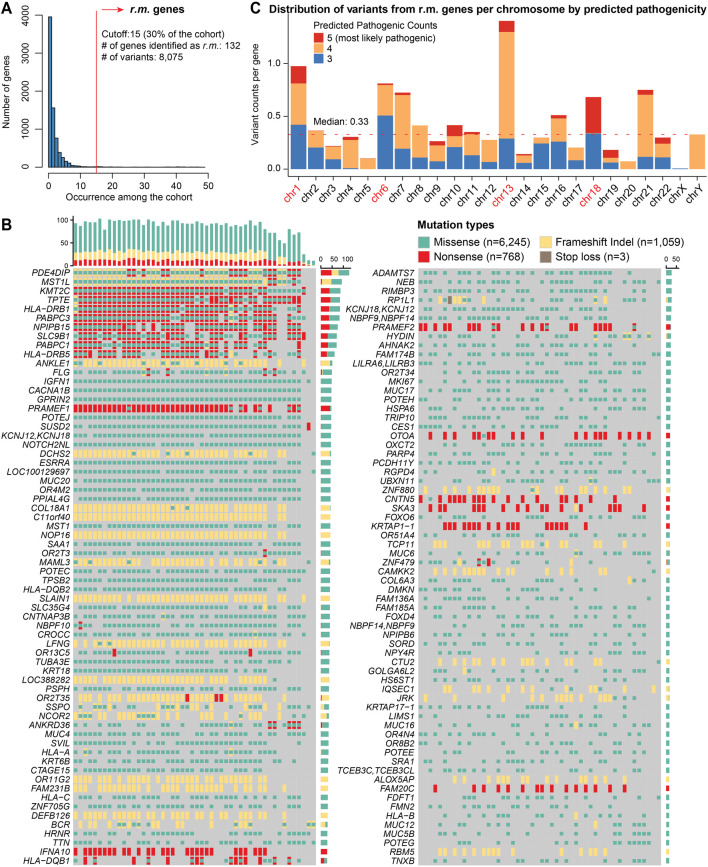
Mutational landscape of *r.m.* genes in the cardiac arrhythmia cohort. **(A)** Histogram representing the frequency of recurrent variants across genes within the arrhythmia cohort. The x-axis indicates the number of times a variant recurs among the cohort, while the y-axis shows the number of genes. A red vertical line marks the cutoff of 15 occurrence, which corresponds to 30% of the cohort and identifies a subset of 132 genes with recurrently mutated (*r.m.*). **(B)** Mutational print heatmap visualizing the mutation types across the 132 *r.m.* genes. Each row represents a gene, and each column represents a patient sample. The different types of mutations are color-coded, with missense (green), nonsense (red), stop loss (brown), and frameshift Indels (yellow). The bars on the mutational print heatmap display the total mutation count per sample (top) and per gene (right). **(C)** Stacked bar plot showing the distribution of variants from *r.m.* genes per chromosome, normalized by the number of protein-coding genes per chromosome. Colors indicate the number of pathogenicity prediction tools (among SIFT, PolyPhen2_HVAR, PolyPhen2_HDIV, MutationTaster, and CADD) that classified each variant as deleterious (5 = most likely pathogenic). The red dotted line represents the median normalized variant density across all chromosomes.

We prioritized genes that were recurrently mutated (*r.m.*) across our arrhythmia cohort. We established a recurrence threshold of 15 (representing 30% of the cohort), and captured genes consistently implicated in cardiac arrhythmia (Figure 2A). This approach revealed 132 *r.m.* candidate risk genes harboring a total of 8,075 variants (Figure 2B; Supplementary Table S2), providing a foundation for understanding the genetic architecture underlying cardiac arrhythmia in our cohort. We noticed several genes exhibited very high mutation frequencies, such as *PDE4DIP*, *MST1L*, *PRAMEF1*, and members of the *HLA* family. The mutation patterns varied considerably across genes, with some displaying primarily missense variants (e.g., *ADAMTS7*, *CACNA1B*) while others showed enrichment for potentially more disruptive frameshift or nonsense mutations (e.g., *IFNA10*, *OTOA*).

Additionally, chromosomal mapping demonstrated non-uniform variant distribution, with significant enrichment on chromosomes 1, 6, and 13 (Figure 2C). The pronounced enrichment on chromosome 6 (variants: n = 822; protein coding genes: n = 1,014) primarily stemmed from extensive mutations in the *HLA* gene family, consistent with previous reports of high polymorphism rates in these immunologically relevant loci (Shiina et al., 2009). The notable peaks on chromosome 1 (variants: n = 1,962; protein coding genes: n = 2,013) corresponded to clusters of genes involved in cell cycle regulation and signal transduction pathways—processes critical for cardiac electrophysiology (Grune et al., 2021) and have been previously implicated in arrhythmogenic processes (Schwartz et al., 2020). Chromosome 13 exhibits the highest normalized density of recurrent variants when adjusted for the number of protein-coding genes (n = 315). Further investigation revealed 442 variants (missense, 284; nonsense, 83; and frameshift indels, 75) distributed across five genes among our cohort (Supplementary Table S2): inflammatory control (*ALOX5AP*), RNA metabolism (*PABPC3*), DNA repair and chromatin remodeling (*PARP4*), and cellular structural integrity (*SKA3*, *SLAIN1*). Moreover, chromosome 18 emerges with a moderate variant count but the highest proportion of deleterious predictions. All those variants were within *SLC35G4*, which encodes a nucleotide sugar transporter involved in glycosylation pathways. Its disruption may affect cardiac glycoprotein processing and cellular signaling networks essential for proper cardiac function.

### 3.3 Functional analysis reveals r.m. genes implicated in immune regulation and ECM components

To assess the clinical and functional relevance of these *r.m.* genes, we first annotated them using established disease databases. For ClinVar (Landrum et al., 2018), only 143 variants (1.77%) returned valid annotations; for OMIM database (Hamosh et al., 2005), 4,700 variants (58.2%) had at least one associated OMIM entry. Two genes harbored variants with established cardiac pathogenicity (Supplementary Table S3): (1) variants in *TTN* were associated with multiple cardiac phenotypes including dilated cardiomyopathy (OMIM: 604145), hypertrophic cardiomyopathy (OMIM: 613765), limb-girdle muscular dystrophy with cardiac involvement (OMIM: 608807), and myopathy with early-onset fatal cardiomyopathy (OMIM: 611705); and (2) variants in *MST1* were associated with immunodeficiency syndromes (OMIM: 614868) that can present with cardiac manifestations. These findings indicate the limited direct overlap between our cohort’s mutational profile and currently catalogued pathogenic variants in cardiac disease databases, suggesting that the genetic landscape of arrhythmia may involve novel or incompletely characterized pathogenic networks. Therefore, to further explore the functional implications of these 132 *r.m.* genes, we performed protein-protein interaction (PPI) (Szklarczyk et al., 2021) and gene ontology (GO) analysis (Wu et al., 2021), revealing critical insights into arrhythmia pathogenesis and its potential progression to cardiomyopathy and cardiac fibrosis.

**FIGURE 3:**
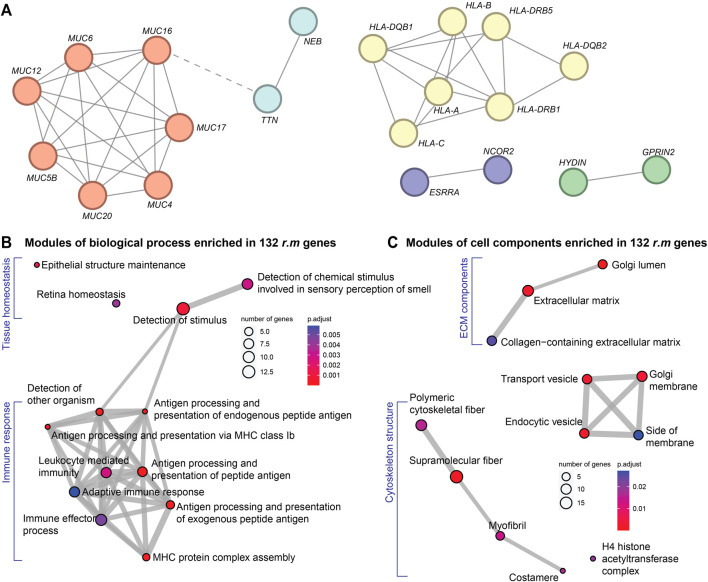
Functional modules of *r.m.* genes in the cardiac arrhythmia cohort. **(A)** PPI network showing the crosstalk among our 132 *r.m.* genes. Node colors reflect STRING-inferred functional clusters. Red indicates mucin family; yellow highlights HLA family; light blue denotes the NEB–TTN pair involved in sarcomere structure; purple marks the ESRRA–NCOR2 pair regulating transcriptional and metabolic pathways; and green represents the HYDIN–GPRIN2 pair, linked to signaling cascades and ciliary function. **(B)** and **(C)** GO enrichment modules of the 132 *r.m.* genes with **(B)** biological process and **(C)** cell components. Node colors indicate FDR-adjusted *P*-values.

PPI network analysis identified two major gene clusters alongside three significant protein pairs (Figure 3A). The first prominent cluster consisted of mucin family genes, which encode glycoproteins critical for maintaining epithelial barrier integrity. Recent evidence suggests that mucin dysregulation affects not only gastrointestinal (Fan et al., 2023) and respiratory (Guo et al., 2023) tract protection but also influences cardiac pathophysiology through gut-heart axis disruption (Fan et al., 2023). Mucin alterations may compromise intestinal barrier function, leading to systemic inflammation and subsequent cardiac electrical remodeling. The second major cluster comprised *HLA* gene families, which orchestrate immune responses and antigen presentation. Variations within these genes are known to modulate the cardiac immune environment, prompting inflammatory reactions that have been recognized as significant precursors to cardiac arrhythmias (Darsee et al., 2010; Li et al., 2021), and ultimately, myocardial fibrosis. Among the identified protein pairs, the *NEB-TTN* interaction is noteworthy. These structural proteins maintain sarcomere integrity and myofibril elasticity; their disruption could compromise mechanical coupling between cardiomyocytes, potentially establishing a substrate for both arrhythmias and progressive fibrotic remodeling (Laitila et al., 2020; Splawski et al., 2005). The *ESRRA-NCOR2* pair regulates metabolic and transcriptional processes essential for cardiac homeostasis, with dysregulation potentially exacerbating oxidative stress and fibroblast activation (Cividini et al., 2021; Van Ouwerkerk et al., 2020). The *HYDIN-GPRIN2* interaction affects cellular signaling cascades that could influence both electrical conduction and fibrotic responses (Brünger et al., 2023; Pierpont et al., 2018).

GO enrichment analysis substantiated these findings (Supplementary Table S4), revealing significant enrichment of immune-related biological processes (Figure 3B) and the involvement of extracellular matrix (ECM) elements (Figure 3C). First, multiple pathways related to antigen processing and presentation were prominently represented, including MHC complex assembly and both endogenous and exogenous peptide antigen processing (Fan et al., 2023; Li et al., 2021). This immune signature suggests that genetic predisposition to aberrant immune responses may trigger chronic inflammation, a recognized driver of electrical instability and subsequent cardiac fibrosis (London, 2017). Cellular component analysis further highlighted significant enrichment in collagen-containing ECM, transport vesicles, and cytoskeletal components. These findings directly implicate ECM remodeling and fibrosis-related processes in arrhythmia pathogenesis. Additionally, enrichment in Golgi apparatus components suggests alterations in protein processing and secretion pathways crucial for ECM maintenance and immune function.

Our functional analyses reveal a genetic framework linking cardiac arrhythmia to immune dysregulation and ECM remodeling. The identified mutations likely create a substrate for both electrical disturbances and structural remodeling, establishing a molecular basis for the observed clinical progression from arrhythmias to cardiomyopathy and cardiac fibrosis in susceptible individuals (Schwartz et al., 2020; Shoureshi et al., 2024; Sohns and Marrouche, 2020; Piek et al., 2019).

### 3.4 Single-cell shows conserved transcriptomic evidence links WEG identified r.m. genes

**FIGURE 4:**
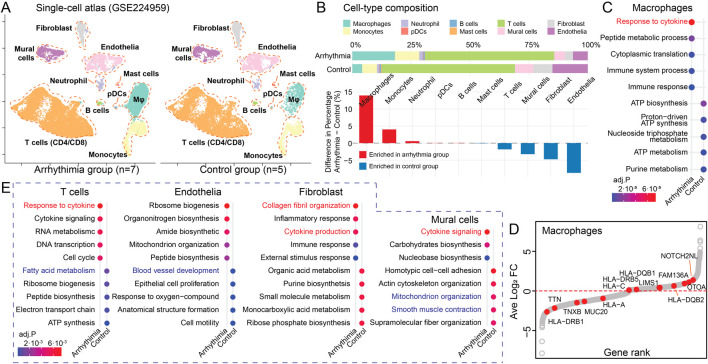
Cell-type-specific Mapping of Recurrent Variant Genes. **(A)** UMAP visualizing the single-cell atlas of an arrhythmia group (n = 7) and a control group (n = 5). **(B)** Cell-type composition differences between arrhythmic and control hearts. Upper panel: Stacked bar plots showing the percentage composition of each cell type in arrhythmic and control conditions. Lower panel: Bar plot displaying the percentage differences between conditions. Red, cell types enriched in arrhythmia; Blue, cell types enriched in control hearts. **(C)** Dot plot showing the enriched pathways in macrophages from arrhythmia versus control groups, colored by adjusted *P*-value. **(D)** Scatter plot illustrating DEGs in macrophages between arrhythmia and control groups. The x-axis shows the gene rank based on log2 fold change, and the y-axis displays the log2 fold change. Red dots highlight the *r.m.* genes. **(E)** Dot plots displaying DE GO terms between arrhythmia and control groups among the rest cell types.

To validate our genetic findings at the transcriptomic level and establish their relevance in specific cardiac cell populations, we analyzed scRNA-seq data from cardiac tissues of arrhythmia patients (n = 7, atrial fibrillation) and healthy controls (n = 5) from a public dataset (Supplementary Table S5) (Hulsmans et al., 2023). Given the consistent enrichment of immune pathways and ECM components among our 132 *r.m.* genes, we hypothesized that their transcriptional consequences would be particularly evident in immune and stromal cell populations. We proceeded with our established pipeline (Huang et al., 2023), and cell type annotation was confirmed by expression of canonical markers (Supplementary Figure S2). This procedure yielded 37,675 cells (arrhythmia, n = 22,532; control, n = 15,143) and identified nine distinct cell types (Figure 4A), including myeloid lineages, neutrophils, mast cells, plasma dendritic cells (pDCs), T cells, B cells, endothelial cells, fibroblasts, and mural cells. Cell-type composition exhibited significant alterations between arrhythmic and control hearts (Figure 4B). Notably, macrophages showed the most pronounced enrichment in arrhythmic samples (+13.0% difference), followed by monocytes (+4.0% difference). Conversely, structural cell type endothelial cells (−8.7% difference) were proportionally reduced in arrhythmic hearts, potentially reflecting tissue remodeling processes.

Macrophage-focused DE analysis revealed that arrhythmia-associated macrophages exhibited upregulated cytokine response and immune activities with concurrent downregulation of metabolic processes (Figure 4C; Supplementary Table S6), consistent with a pro-inflammatory phenotype. When mapping our 132 *r.m.* genes onto macrophage DE results (Figure 4D), we observed significant upregulation of *NOTCH2NL* and *HLA* family genes, which are reported to drives inflammation and fibrosis (Fiddes et al., 2018). Conversely, *TNXB*, an ECM glycoprotein that regulates collagen fibrillogenesis, showed decreased expression (Schalkwijk et al., 2001). This observed pattern suggests a shift toward pro-inflammatory states with altered ECM regulation in arrhythmia-associated macrophages.

Our comparative analysis of differentially enriched pathways across other major cell types revealed a conserved signature of elevated cytokine responses (Figure 4E; Supplementary Figure S3; Supplementary Table S6), underscoring a potential shared inflammatory mechanism driven by *r.m.* genes. Additionally, we identified cell-type-specific dysfunctions: T cells showed reduced fatty acid metabolism; endothelial cells exhibited compromised vasculogenesis; fibroblasts displayed increased collagen fibril organization; and mural cells demonstrated alterations in mitochondrial organization and smooth muscle contraction. The fibroblast-specific enhancement of collagen organization aligns with the ECM remodeling pathways identified in our genetic analysis, further supporting the role of fibrotic processes in arrhythmogenesis.

### 3.5 The contributing role of r.m. genes to SPP1+ macrophage-mediated inflammation and ECM remodeling

**FIGURE 5:**
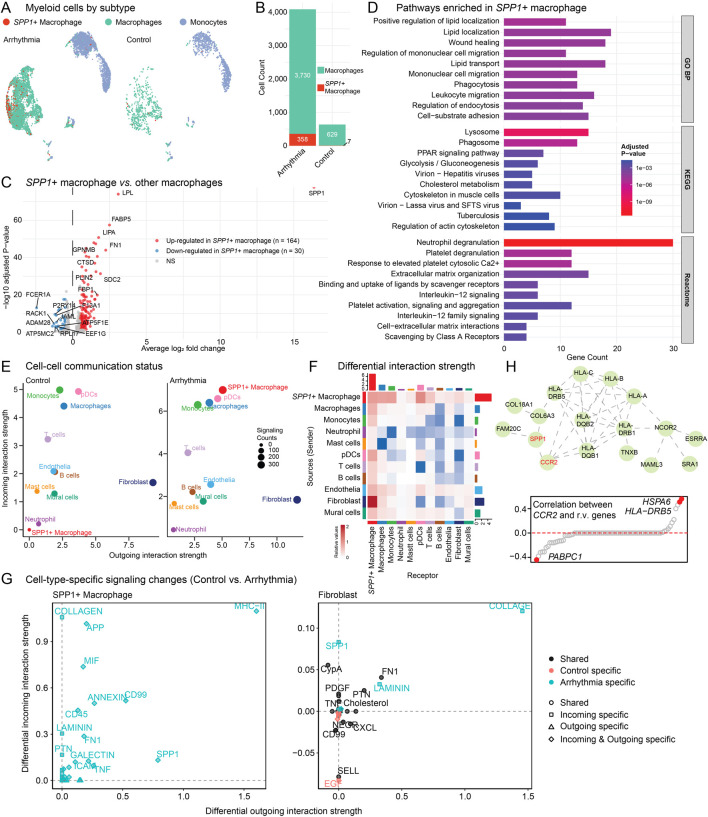
SPP1+ macrophage characterization and cell-cell communication analysis. **(A)** UMAP visualization of myeloid cell subtypes. *SPP1*+ macrophages are mainly enriched in arrhythmic hearts. **(B)** Bar plot quantifying *SPP1*+ macrophage and total macrophage cell counts between arrhythmic and control groups. **(C)** Volcano plot showing DE genes of *SPP1*+ macrophages versus other macrophages. **(D)** Barplot showing pathway enrichment analysis of *SPP1*+ macrophage-specific genes using GO:BP, KEGG and Reactome database. **(E)** Cell-cell communication analysis using CellChat showing interaction strength. Circle size represents signaling counts. **(F)** Heatmap displaying differential cell-cell interaction patterns. Red, increased in arrhythmia group; Blue, enriched in control group. **(G)** Cell-type-specific signaling pathway changes between control and arrhythmic conditions. Left panel shows SPP1+ macrophage. Right panel shows fibroblast cells. **(H)** PPI network (top) showing the crosstalk between *SPP1*/*CCR2* genes and the 132 *r.m.* genes. Scatter plot (bottom) showing the correlation between *CCR2* and the 132 *r.m.* genes in macrophages.

Previous research has established that *SPP1+* macrophages play a critical role in cardiac arrhythmogenesis (Hulsmans et al., 2023). Importantly, the *SPP1* protein functions as a multifaceted signaling molecule with dual roles in promoting both inflammation and fibrosis, perfectly aligning with the immune dysregulation and ECM remodeling pathways identified in our genetic analysis. To further characterize this critical cell subpopulation, we first identified *SPP1*+ macrophages (Figure 5A; Supplementary Figure S4). *SPP1*+ macrophages were markedly enriched in arrhythmic hearts (Figure 5B), indicating that the observed *SPP1* signal reflects specific cellular infiltration rather than broad transcriptional upregulation across the macrophage population (Hulsmans et al., 2023).

DE analysis comparing *SPP1*+ macrophages to other macrophages revealed 164 upregulated genes (Figure 5C; Supplementary Table S7). Key markers include *SPP1*, *FABP5* (fatty acid binding protein), *LIPA* (lysosomal acid lipase), and *FN1* (fibronectin 1, ECM glycoprotein). Those makers indicate a metabolically active population involved in lipid processing, lysosomal function, fibrotic processes, and ECM remodeling. Pathway enrichment analysis of *SPP1*+ macrophage-specific genes revealed a complex functional profile (Figure 5D): while these cells exhibited classical M2-like macrophage signatures (Yunna et al., 2020) (e.g., lipid localization and transport, phagocytosis, lysosomal function, and wound healing), they simultaneously displayed pro-inflammatory characteristics through IL-12 signaling and neutrophil degranulation pathways. Additionally, prominent enrichment in extracellular matrix organization and cell-substrate adhesion pathways underscores their role in tissue remodeling processes. This mixed polarization profile suggests that *SPP1*+ macrophages represent a distinct pathological subset that combines tissue remodeling capacity with sustained inflammatory activity (Reggio et al., 2025; Palma, 2025), consistent with their proposed role in driving both immune dysregulation and fibrotic remodeling in cardiac arrhythmia.

To systematically investigate the communication networks involving *SPP1*+ macrophages, we inferred cell-cell interaction using CellChat (Jin et al., 2025) (Figures 5E–G). The *SPP1*+ macrophages undergo remarkable activation from normal to disease conditions, showing enhanced both outgoing and incoming signaling strength compared to other cell types (Figure 5E). This positions *SPP1*+ macrophages as central signaling hubs in the arrhythmic cardiac microenvironment. Comprehensive signaling pathway details across all cell types are provided in Supplementary Figure S5 (arrhythmia hearts) and Supplementary Figure S6 (normal controls). Next, we performed differential interaction strength analysis between conditions. *SPP1*+ macrophage-fibroblast communication is significantly enhanced in arrhythmic hearts (Figure 5F). Motivated by this observation, we examined the specific signaling pathways mediating *SPP1*+ macrophage-fibroblast crosstalk in detail (Figure 5G). *SPP1*+ macrophages showed enhanced outgoing signals including COLLAGEN, APP (amyloid precursor protein), MIF (macrophage migration inhibitory factor), and *SPP1* pathways, all exclusively upregulated in arrhythmic conditions. Correspondingly, fibroblasts exhibited increased incoming COLLAGEN and SPP1 signaling, along with enhanced FN1 (fibronectin) and LAMININ pathways. This bidirectional enhancement creates a comprehensive network of inflammatory and ECM remodeling signals, establishing a potential positive feedback loop where activated fibroblasts may further recruit *SPP1*+ macrophages, perpetuating both inflammatory and fibrotic processes in arrhythmic hearts.

Another key question in arrhythmia pathogenesis is what genetic mechanisms drive the recruitment and activation of *SPP1*+ macrophages. Previous studies identified elevated *CCR2* as critical for *SPP1*+ macrophage recruitment (Hulsmans et al., 2023), but the genetic underpinnings remained unclear. We hypothesized that dysregulated *r.m.* genes could activate the *CCR2/SPP1* axis, thereby facilitating inflammatory macrophage infiltration while simultaneously promoting fibrotic remodeling. Our PPI network analysis revealed direct interactions between the *CCR2/SPP1* axis and multiple *r.m.* genes (Figure 5H, top), particularly with the *HLA* gene family, which showed elevated expression in arrhythmic conditions (Supplementary Figure S3). Notably, *SPP1* also displayed interactions with *COL18A1* and *COL6A3*, two *r.m.* genes upregulated in fibroblasts (Supplementary Figure S3), establishing a molecular bridge between macrophage-driven inflammation and cardiac fibrosis (Zhang et al., 2019). We further explored *CD44*, another key regulator of *SPP1*+ macrophages (Palma, 2025), and found extensive PPI connections within *r.m.* genes including additional ECM components (Supplementary Figure S7). This suggests a potential mechanism through which *SPP1*+ macrophages may directly mediate inflammatory-fibrogenic network, two critical hallmarks in arrhythmia progression and persistence (Shoureshi et al., 2024; Sohns and Marrouche, 2020). Correlation analyses within macrophages confirmed positive associations between *CCR2* and the *r.m.* genes *HLA-DR5* and *HSPA6* (Figure 5H, bottom), key players in immune modulation and stress response (Grune et al., 2021; London, 2017). The negative correlation with *PABPC1*, involved in mRNA stability, suggested translational stress in arrhythmia-associated macrophages, further supported by concurrent *HSPA6* upregulation (Maxwell et al., 2021).

In summary, our single-cell analysis offers transcriptomic validation of our genetic findings and provides mechanistic insights into how *r.m.* genes might drive cardiac arrhythmia through dual inflammatory and fibrotic pathways. The *SPP1*+ macrophage emerges as a central cellular mediator, linking immune dysregulation and ECM remodeling—the two key pathways identified in our *r.m.* gene functional analysis. These findings suggest that genetic variants in immune and ECM-related genes may predispose certain arrhythmia patients to develop cardiomyopathy through SPP1+ macrophage-mediated inflammation and fibrosis.

### 3.6 Exploratory analysis of clinical associations and mutational hotspots

**FIGURE 6:**
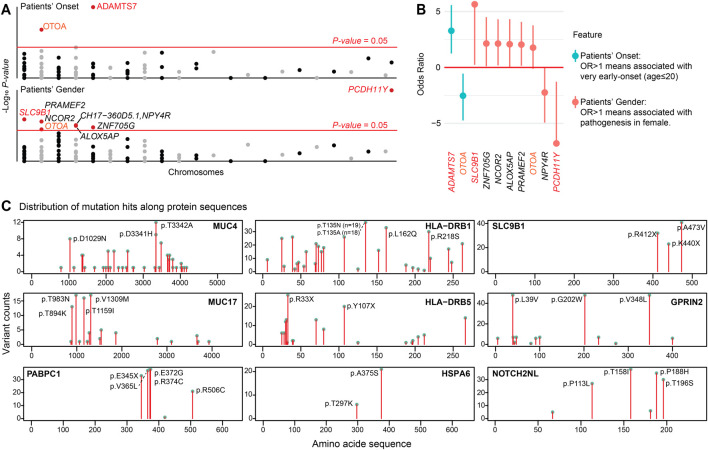
Clinical associations of *r.m.* genes and conserved mutational hotspots. **(A)** Manhatton plot illustrating the association between patient’s onset age and gender with *r.m.* genes. The x-axis represents chromosomes, and the y-axis shows the -log10 P-value for each gene. The red horizontal line indicates the *P-value* = 0.05. **(B)** Forest plot displaying the odds ratio (OR) for the association of patient’s onset age (very early-onset, age ≤20 years) and gender (female) with the presence of specific *r.m.* genes. **(C)** Lollipop plots displaying the frequency and distribution of mutation sites along the amino acid sequences of selected proteins implicated in cardiac arrhythmias. Each plot represents a different protein, with the x-axis indicating the amino acid position within the protein sequence and the y-axis showing the count of variants observed at each position in the study population. Notable mutational hotspots are annotated with the specific amino acid change. These graphical representations elucidate the prevalence of specific mutations, suggesting areas within these proteins that may have critical roles in the pathogenesis of cardiac arrhythmia due to altered protein function.

Building on our pathway and transcriptomic findings, we conducted exploratory analyses to investigate associations between *r.m.* genes and clinical features (Figures 6A,B), and to identify conserved mutational hotspots that might contribute to arrhythmogenesis (Figure 6C). We aimed to complement our WES and single-cell insights, providing additional molecular insights into how specific mutations within *r.m.* genes might contribute to cardiac arrhythmia through disruption of immune regulation and ECM homeostasis.

We identified several significant genotype-phenotype associations. Notably, *ADAMTS7* mutations were enriched in very early-onset arrhythmia patients (OR = 9.71 [2.38–47.74], *P-value* <0.001). *ADAMTS7* encodes a secreted metalloproteinase recognized as a risk locus for coronary atherosclerosis (Bengtsson et al., 2017; Mizoguchi et al., 2021). These mutations likely alter ECM composition and affect vasculature behavior (Mizoguchi et al., 2021), potentially accelerating arrhythmia development. Gender-specific associations revealed that *SLC9B1* mutations, which encode a sodium-hydrogen exchanger regulating cellular pH, were exclusively found in female patients (*P-value* = 0.017). These variants may disrupt intracellular pH balance and consequently affect cardiac electrical activity (Patel et al., 2023). The presence of mutated *SLC9B1* only in females may be influenced by sex-specific genetic expression and hormonal interactions, potentially associated with estrogen signaling (*ESRRA* is recurrently mutated in our cohort), which can modify cardiac ion channel functionality (Van Ouwerkerk et al., 2020). Conversely, *PCDH11Y*, located on chromosome Y, was exclusively identified in male patients (*P-value* <0.001). Interestingly, *OTOA* mutations showed associations with both later onset (OR = 0.17 [0.04–0.68], *P-value* = 0.009) and female predominance (OR = 3.41 [0.92–13.58], *P-value* = 0.045). The age-association parallels observations in *OTOA*-related hearing loss (Sugiyama et al., 2019), possibly reflecting age-dependent expression patterns, while gender-specific regulation may underlie increased female susceptibility to *OTOA* variants. We did not identify genes enriched in patients with concurrent cardiomyopathy (n = 9), either due to limited statistical power from the small subgroup or suggesting arrhythmia patients may share a conserved genetic predisposition toward structural heart disease.

Our analysis further revealed domain-specific mutational hotspots in several key *r.m.* genes (Figure 6C). In mucin family genes (*MUC4*, *MUC17*), mutations predominantly affected extracellular serine/threonine-rich tandem repeats, potentially disrupting glycosylation patterns critical for mucosal barrier function. *PABPC1* harbored mutations within RNA recognition motifs that could compromise mRNA stability and protein synthesis (Maxwell et al., 2021)—consistent with the translational stress observed in our single-cell analysis. In HLA family genes (*HLA-DRB1*, *HLA-DRB5*), mutations clustered in peptide-binding regions, potentially altering antigen presentation capacity and immune responses. *HSPA6* mutations affected domains essential for ATPase activity, potentially impairing stress response functions (Maxwell et al., 2021)—particularly relevant given its correlation with *CCR2* in arrhythmia-associated macrophages. *NOTCH2NL* displayed mutations in EGF-like domains critical for Notch signaling (Fiddes et al., 2018). *SLC9B1* mutations concentrated in transmembrane domains, potentially disrupting ion transport (Patel et al., 2023), while *GPRIN2* mutations might interfere with G protein-coupled receptor interactions affecting downstream signaling (Brünger et al., 2023; Pierpont et al., 2018).

## 4 Discussion

Our exploratory study provides novel insights into the complex interplay between genetics variants and cellular transcriptional changes in cardiac arrhythmias. From our cohort of 50 individuals, we identified 132 *r.m.* genes that consistently appeared across patients and showed significant enrichment in pathways involved in immune regulation, extracellular matrix composition, and tissue homeostasis. This conserved genetic pattern suggests shared molecular mechanisms underlying cardiac arrhythmia patients despite clinical heterogeneity. We also observed consistent transcriptional alterations across various cell types at the single-cell level, marked by enhanced cytokine response, immune activation, and fibrogenic signaling, further support our hypothesis of a shared genetic basis driving these pathological changes. Several of our identified *r.m.* genes may establish a genetic susceptibility for enhanced *SPP1*+ macrophage recruitment and activation—a cellular mechanism previously observed in arrhythmias but whose genetic basis remained unclear. Additionally, the prevalence of mutations in the *HLA* gene family underscores the significant role of inflammatory processes in arrhythmia pathogenesis (Hulsmans et al., 2023; London, 2017). The mutations in the *MUC* gene family open new research and treatment avenues by suggesting a potential link between the gut microbiome and cardiac health (Fan et al., 2023; Witkowski et al., 2020).

**FIGURE 7:**
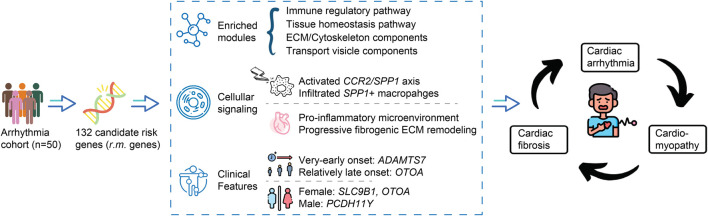
Proposed integrative model linking *r.m.* genes to arrhythmia-cardiomyopathy-fibrosis progression. Schematic representation of the proposed mechanistic framework connecting genetic variants to cardiac disease progression. Starting from our arrhythmia cohort (n = 50), we identified 132 candidate risk genes (*r.m.* genes) enriched in modules related to immune regulation, tissue homeostasis, ECM/cytoskeleton components, and transport vesicle functions. These genetic variants appear to influence cellular signaling pathways, particularly the *CCR2/SPP1* axis, inducing the infiltration of *SPP1+* macrophages. The resulting cellular dysfunction manifests as a pro-inflammatory microenvironment and progressive fibrogenic ECM remodeling. Clinical associations revealed age-related patterns (*ADAMTS7* with very-early onset, *OTOA* with relatively late onset) and gender-specific variants (*SLC9B1* and *OTOA* in females, *PCDH11Y* in males). This integrated model suggests a potential vicious cycle where genetic predisposition may drive not only electrical disturbances (arrhythmia) but also structural abnormalities (cardiomyopathy) and tissue remodeling (cardiac fibrosis), with each component potentially reinforcing the others through sustained inflammatory and fibrotic processes.

The integration of genetic and transcriptomic findings allows us to propose an integrative model that may guide future investigations (Figure 7). In this model, genetic variants within the identified *r.m.* genes could potentially alter protein expression or function, particularly at the mutational hotspots we observed. These alterations might influence the *CCR2/SPP1* axis while simultaneously affecting ECM composition and homeostasis. The resulting dual impact—a pro-inflammatory environment characterized by elevated cytokine responses alongside aberrant ECM remodeling—might contribute to both electrical disturbances and progressive fibrotic changes, potentially explaining the observed progression from arrhythmias to cardiomyopathy in a subset of patients.

Beyond arrhythmia, accumulating evidence highlights *SPP1+* macrophages as central mediators across diverse cardiac pathologies (Palma, 2025; Hoeft et al., 2023; Kuppe et al., 2022; Jung, 2022). In human heart failure (Hoeft et al., 2023), single-cell transcriptomic profiling revealed a marked expansion of *SPP1+* macrophages, co-expressing markers such as *FN1* and *TREM2*, and occupying fibrotic niches where they orchestrate ECM remodeling through *CXCL4* signaling. Similarly, spatial and single-cell analyses (Kuppe et al., 2022; Jung, 2022) in human myocardial infarction tissue demonstrate accumulation of *SPP1+* macrophages at infarct borders, where they closely interact with myofibroblasts to influence scar formation and remodeling. Thus, *SPP1+* macrophages are further emphasize in manifesting conserved fibrotic, immunomodulatory, and ECM-remodeling phenotypes across organs, often correlating with poor outcomes and meriting classification as a distinct macrophage subtype (Reggio et al., 2025; Palma, 2025).

We acknowledge several limitations in our study. First, while our WES cohort size is modest, we intentionally recruited younger patients (≤35years) to minimize non-genetic confounders, enabling us to detect both conserved genetic patterns despite the limited sample size. Second, our WES cohort (Chinese Han population) and scRNA-seq data (North American atrial fibrillation patients) come from different populations, potentially introducing population-specific confounders (Guo et al., 2023); however, the consistency of our findings across these diverse populations actually strengthens the argument for conserved pathogenic mechanisms. Third, while we identified overlapping genetic factors across arrhythmia subtypes, we recognize that the AF-skewed single-cell transcriptional profiles may overrepresent atrial-specific pathways rather than universal arrhythmogenic mechanisms. Fourth, our proposed model requires validation in larger cohorts and functional studies, though it is grounded in established literature on inflammatory and fibrotic mechanisms in cardiac disease. Nevertheless, we believe our findings provide a valuable direction for investigating the interplay between genetic predisposition, inflammation, and fibrosis in arrhythmogenesis.

In conclusion, our study provides a foundation for future investigations into the genetic and cellular mechanisms of cardiac arrhythmias. It emphasizes the need for a holistic approach to understanding and treating this complex condition, incorporating genetic, cellular, and systemic factors. Understanding these pathways may eventually enable the identification of patients at higher risk for developing structural heart disease and open avenues for targeted interventions that address both electrical and structural aspects of cardiac pathology.

## Data Availability

Publicly available datasets were analyzed in this study. This data can be found here: https://www.ncbi.nlm.nih.gov/geo/query/acc.cgi?acc=GSE224959.
