# Supplementary material for: Genetic predisposition to immune dysregulation and extracellular matrix remodeling in cardiac arrhythmia reveals potential mediation by SPP1+ macrophages
Source: Front Cell Dev Biol. 2025 Aug 18;13:1611663. doi: 10.3389/fcell.2025.1611663 (PMC12399913; doi:10.3389/fcell.2025.1611663)
Supplement: Supplementary file 2 [file DataSheet1.docx]

**Supplementary Tables and Figures of**

**“**Genetic predisposition to immune dysregulation and extracellular matrix remodeling in cardiac arrhythmia reveals potential mediation by *SPP1*+ macrophages**”**

Jie-Yuan Jin^1,2#^, Shuai Guo^2#^, Yao Deng^3^, Ya-Qin Chen^4^, Chen Liang^5^, Yu-Jie Jiang^2^, Wang Zhao^4*^, Rong Xiang^2^*

^1^ School of Medicine, Shaoxing University, Shaoxing, 312000, China;

^2^ School of Life Sciences, Central South University, Changsha, 410013, China;

^3^ Department of Cardiovascular Surgery, Xiangya Hospital, Central South University, Changsha, 410008, China;

^4^ Department of Cardiovascular Medicine, the Second Xiangya Hospital, Central South University, Changsha, 410011, China;

^5^ Center for Medical Genetics, Jiangmen Maternal & Child Health Care Hospital, Jiangmen, 529000, China.

^#^ Jie-Yuan Jin and Shuai Guo contributed equally to this work.

^*^ Corresponding Authors.

**Supplementary Tables 1-7:** <https://1drv.ms/x/s!Ati0XzrZojHZgZsZdtMQd9C2cjy_7w?e=5FO8ka>

**Table of contents:**

Table S1. Detailed clinical information

Table S2. 132 r.v. genes across patient cohort

Table S3. Catalogued pathogenic variants in cardiac disease databases

Table S4. GO results of 132 r.v. genes

Table S5. Patient demographics and clinical information (scRNA-seq cohort from GSE224959).

Table S6. Cell-type-specific GO results of DE genes between Controls and Arrhythmias

Table S7. Signature genes of SPP1+ macrophage

**Supplementary Figures:**


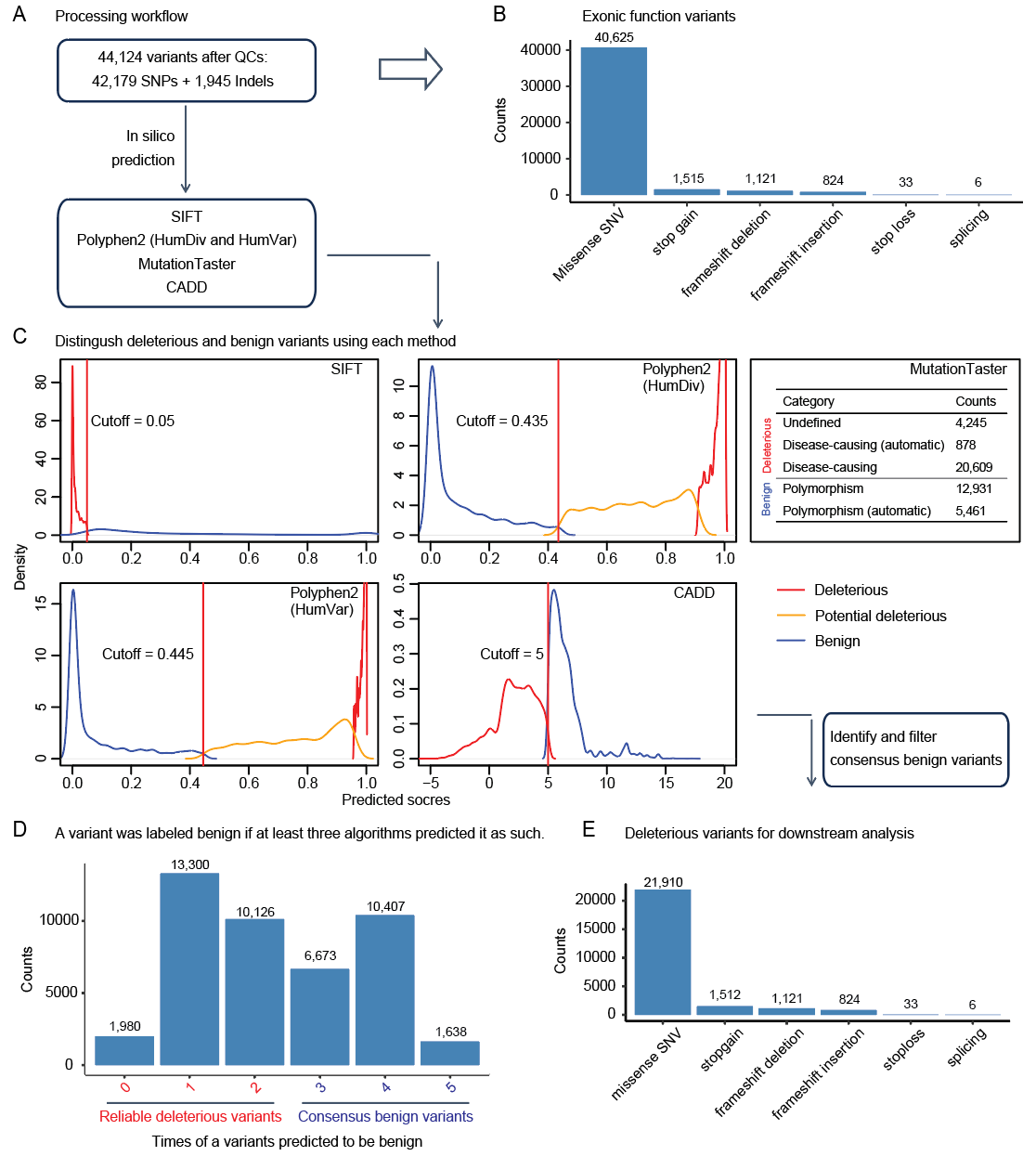


**Figure S1. Variants Filtering and Classification Workflow.**

**(A)** The processing workflow for variant analysis post-quality control screening, illustrating the initial count of variants which includes single nucleotide polymorphisms (SNPs) and insertions/deletions (Indels). Four in-silico tools (SIFT, Polyphen2 (HumDiv and HumVar), MutationTaster, and CADD) are used for a robust variant pathogenicity prediction. (B) Counts of exonic function variants are categorized into missense SNV, stop gain, frameshift deletion, frameshift insertion, stop loss, and splicing. **(C)** Graphs representing the distribution of predicted scores for the variants. According to the cutoff thresholds, variants are classified as deleterious, potentially deleterious, or benign. Red lines indicate the cutoff used to distinguish benign variants. **(D)** Bar graph showing the counts of variants classified based on the consensus of at least three algorithms predicting them as such, differentiating between reliable deleterious variants and consensus benign variants. **(E)** Final counts of deleterious variants that were selected for downstream analysis after the filtering process, broken down by variant type.


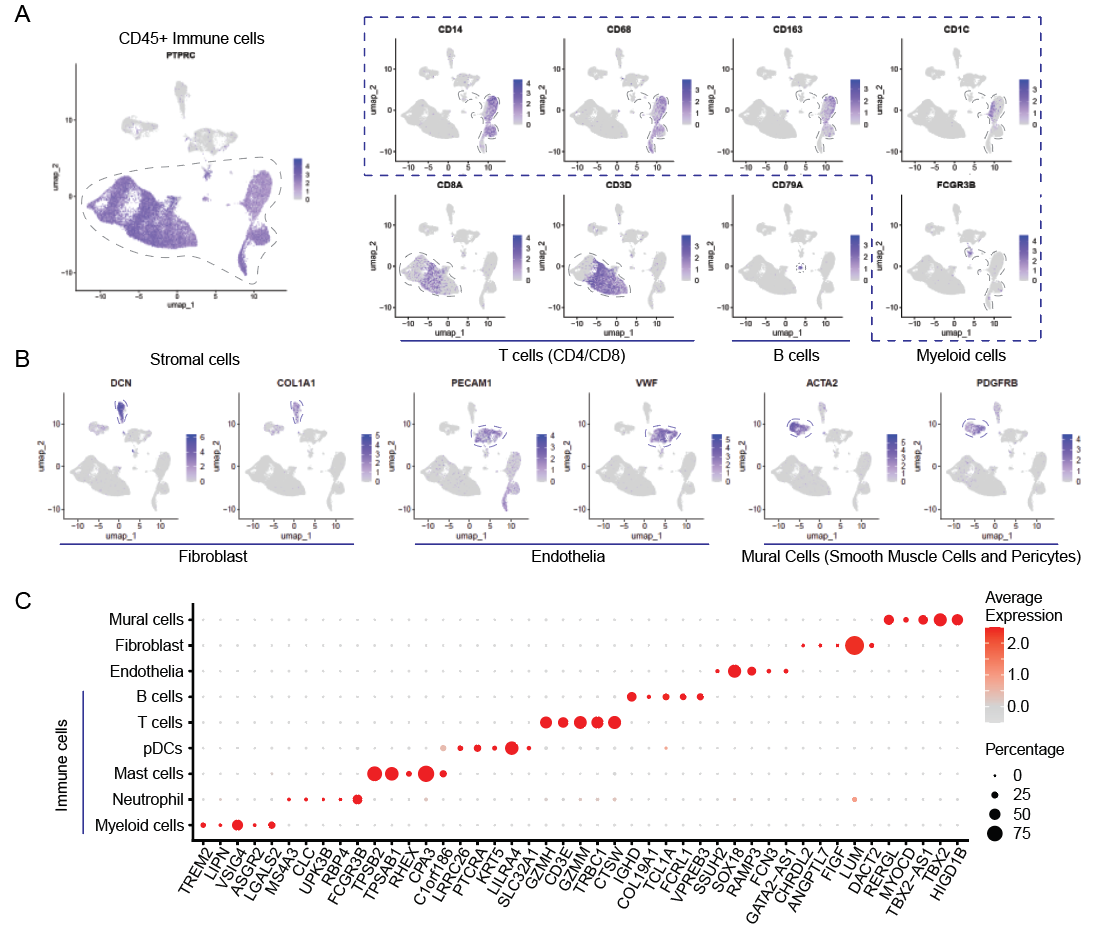


**Figure S2. Annotation of the Human Cardiac Arrhythmias Single-Cell Atlas**

**(A)** UMAP visualization of CD45+ immune cells within the scRNA-seq data, with each panel showing the density of specific cell markers (*PTPRC* for circulating immune cells; *CD14, CD68, CD163,* and *CD16* for myeloid lineages; *CD8A* and *CD3D* for T cells; *CD79A* for B cells). **(B)** UMAP visualization of stromal cells, each highlighted by key cellular markers (*DCN* and *COL1A1* for fibroblast; *PECAM1* and *VWF* for endothelia; *ACTA2* and *PDGFRB* for mural cells). **(C)** Dot plot matrix displaying the expression levels and percentages of various markers across all the annotated cell types. Dot size represents the percentage of cells expressing the marker, and color intensity indicates the average expression level.


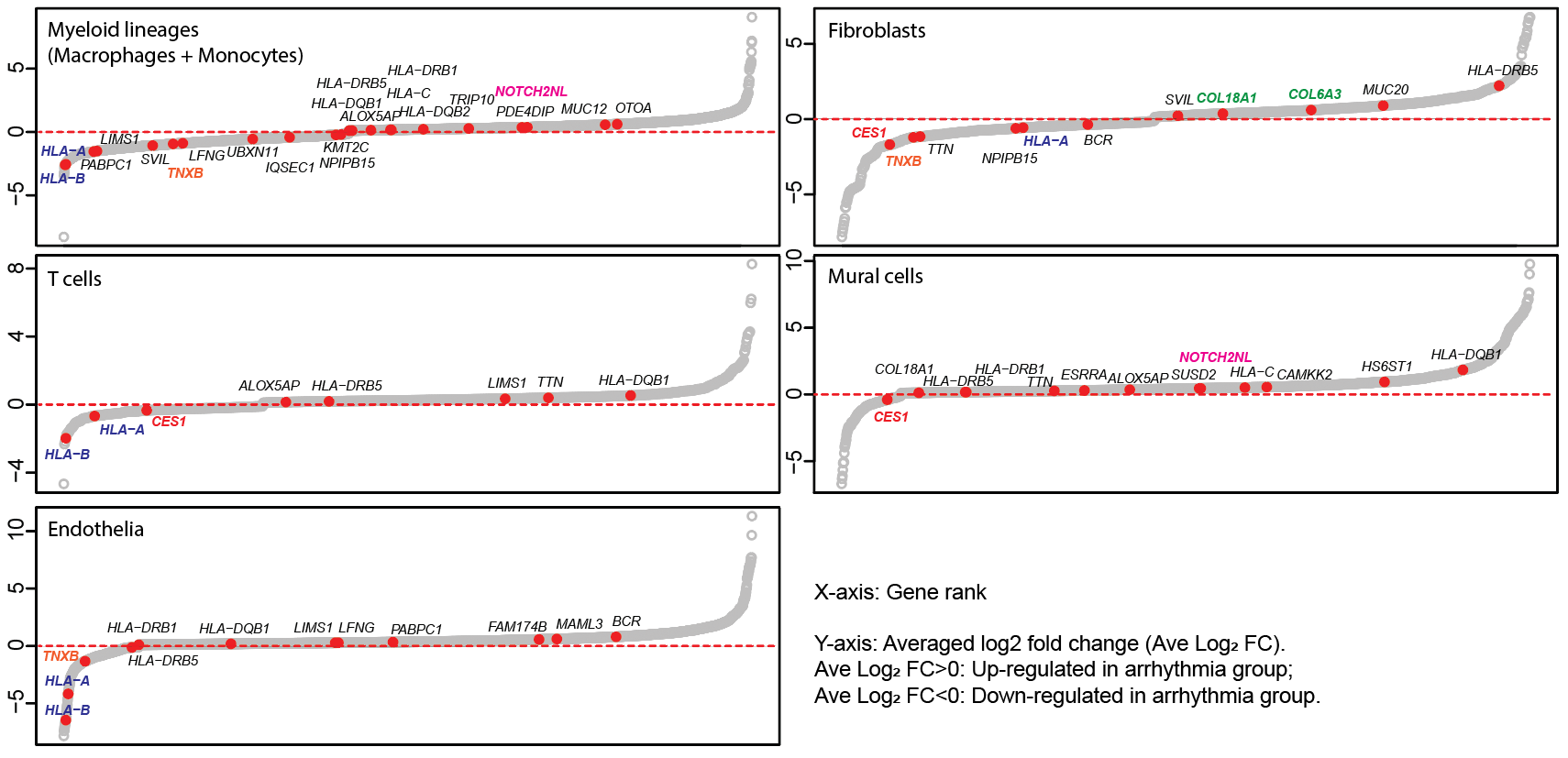


**Figure S3. Recurrent Variant Genes in Cell-type-specific Differential Gene Expression**

Scatter plots for each cell type (myeloid lineages, T cells, endothelia, fibroblasts, and mural cells) displaying differential gene expression between cardiac arrhythmia and healthy control groups. The x-axis represents the gene rank based on the log2 fold change, while the y-axis indicates the specific log2 fold change for each gene. Red dots represent r.v. genes that are significantly upregulated (above the dashed line) or downregulated (below the dashed line) in the arrhythmia group compared to controls.


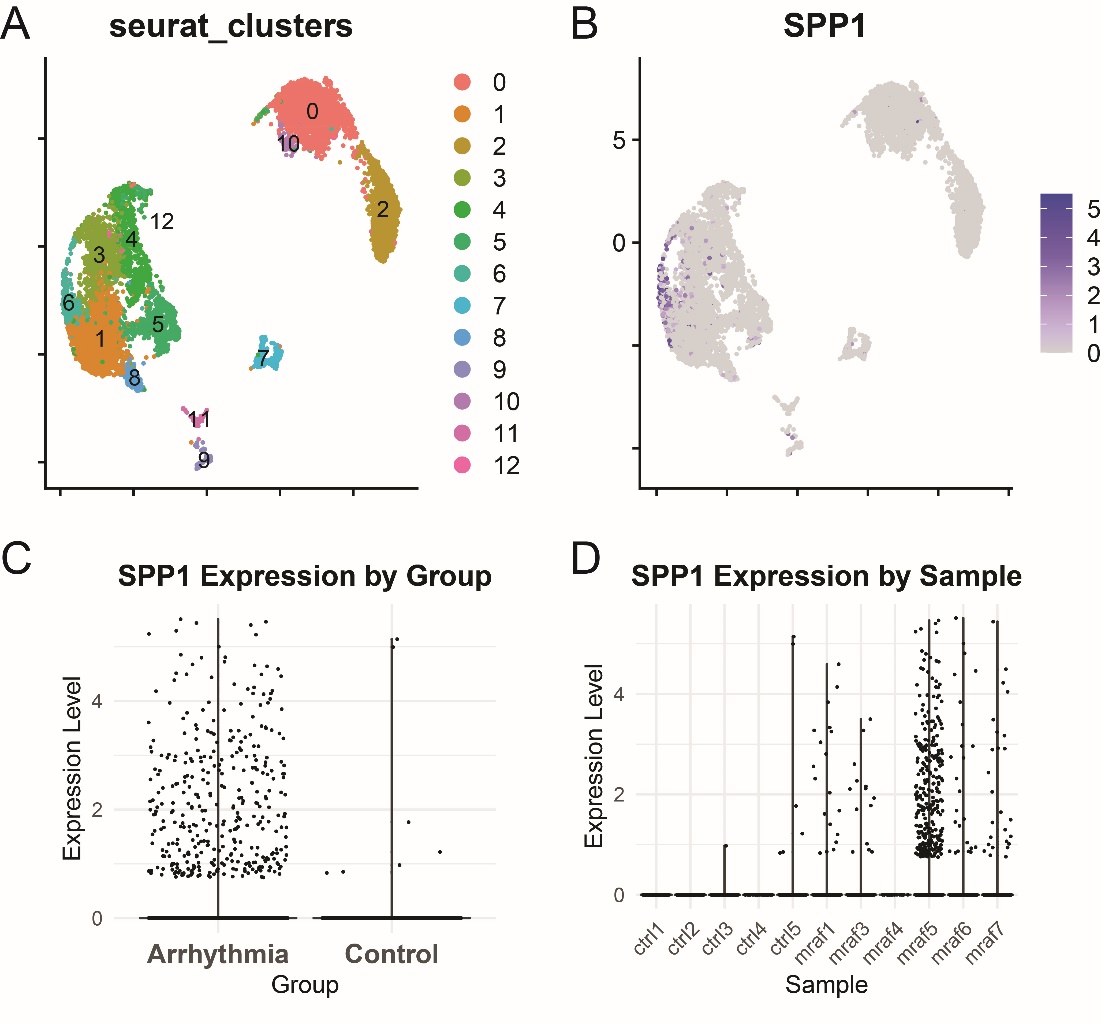


**Figure S4. *SPP1* expression analysis for macrophage subtype identification**

**(A)** UMAP visualization showing Seurat clustering of myeloid lineages. **(B)** *SPP1* gene expression on the same UMAP, with color intensity indicating expression levels. Highest SPP1 expression is observed in Cluster 6. **(C)** Violin plot comparing *SPP1* expression levels between arrhythmic and control groups. Each dot represents a single cell. **(D)** Sample-wise *SPP1* expression distribution.


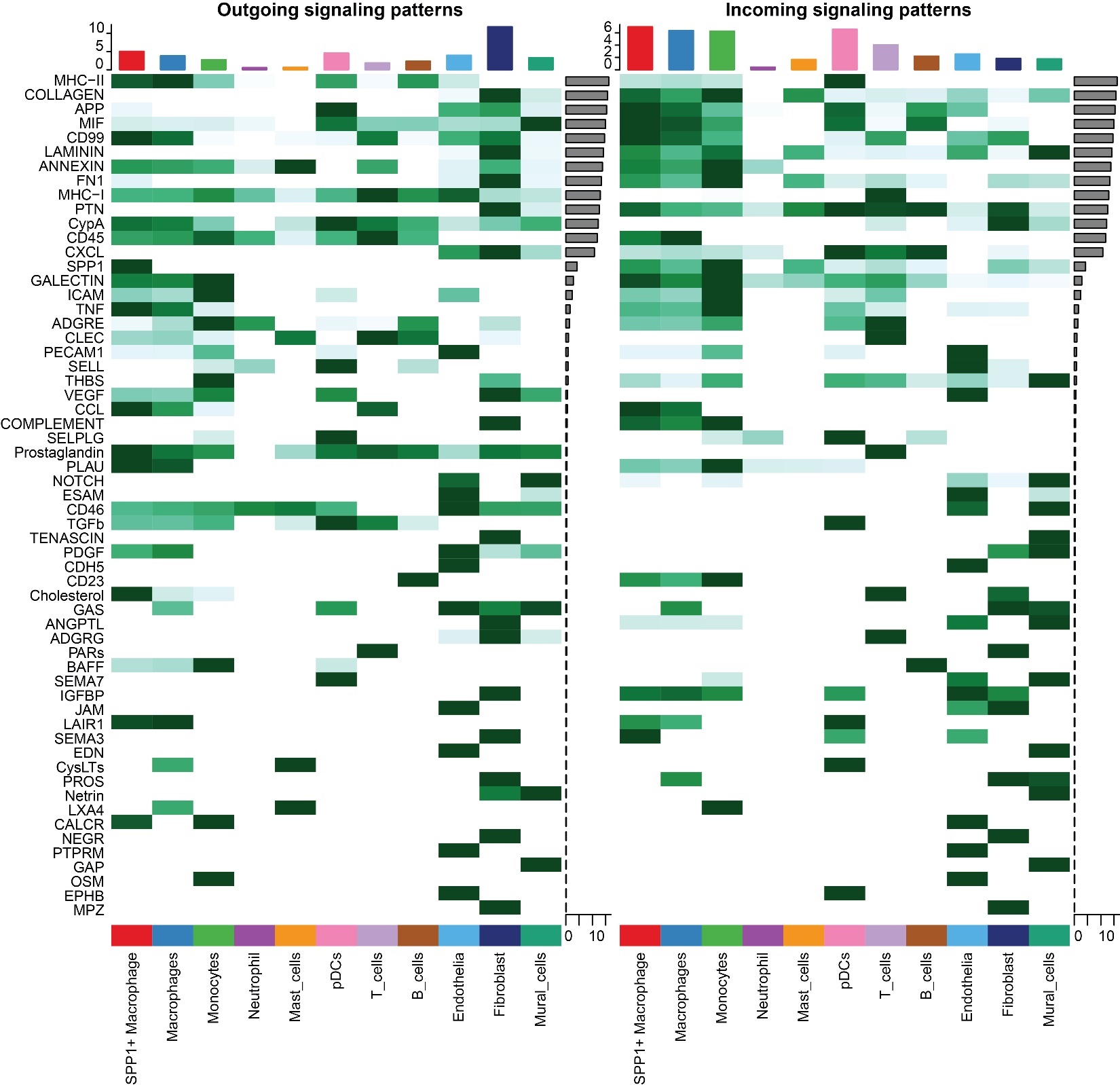


**Figure S5. Cell-cell communication signaling pathways in arrhythmia**


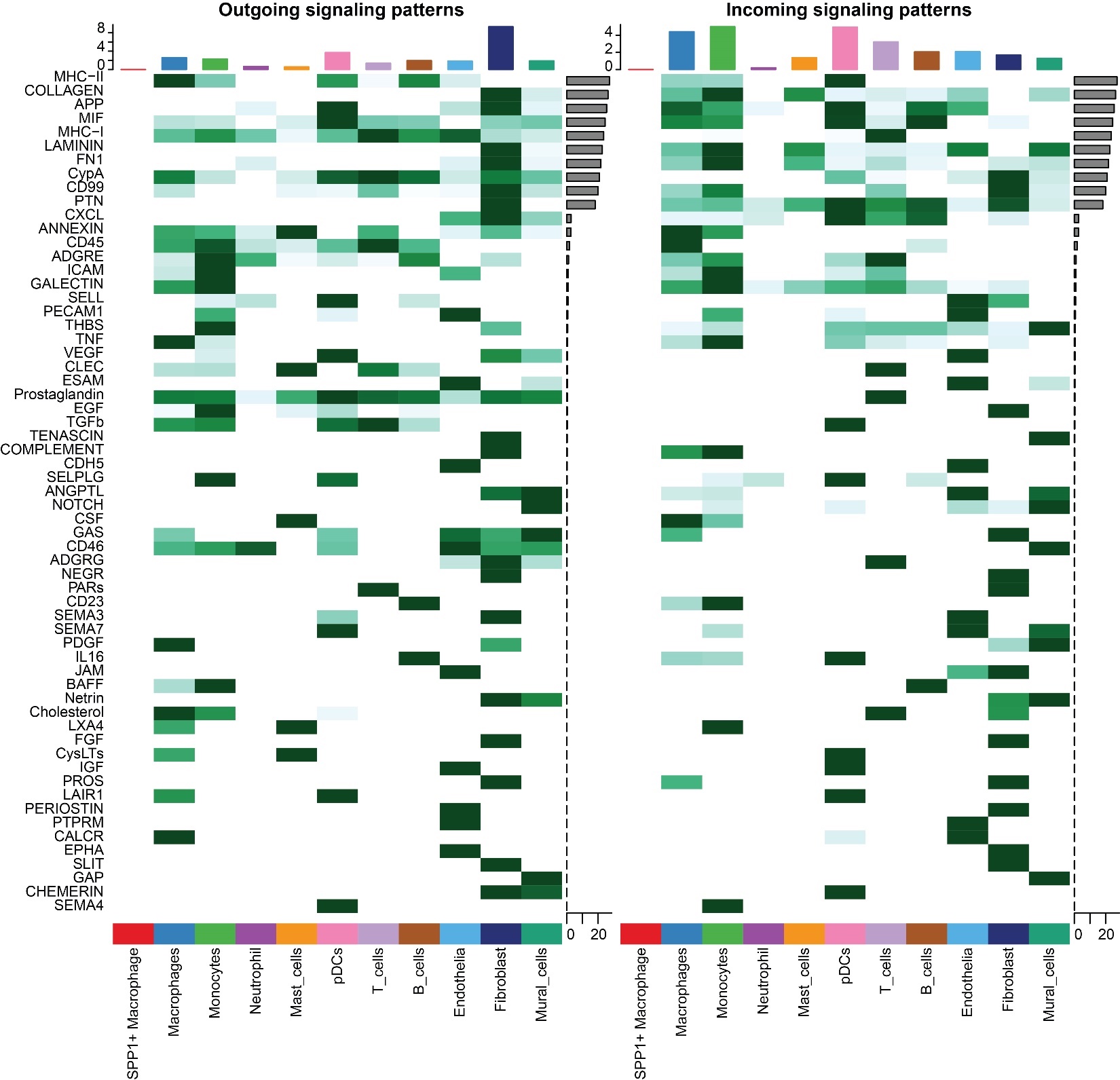


**Figure S6. Cell-cell communication signaling pathways in control**


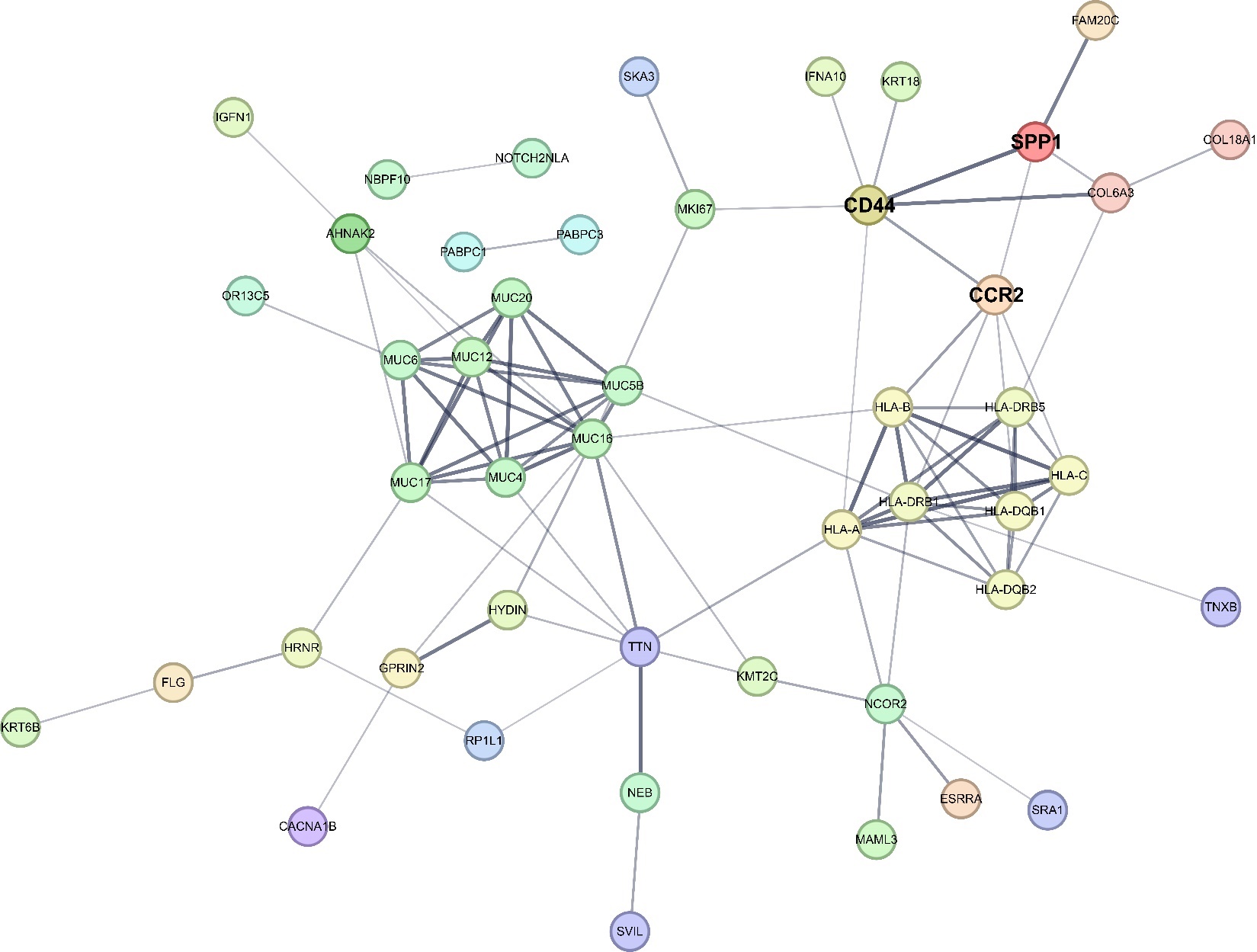


**Figure S7. Protein-protein interaction network of r.m. genes with *SPP1/CCR2/CD44* axis**

Updated PPI network incorporating CD44 alongside SPP1 and CCR2, showing direct and indirect interactions with 132 r.m. genes. Key functional clusters include: mucin families (MUC4, MUC6, MUC12, MUC17, MUC20), HLA families (HLA-A, HLA-B, HLA-C, HLA-DRB1, HLA-DRB5, HLA-DQB1, HLA-DQB2), and structural proteins (TTN, NEB). The central *SPP1/CCR2/CD44* axis connects to collagens (COL18A1, COL6A3) and transcriptional regulators (NCOR2, ESRRA), establishing molecular bridges between immune dysregulation and ECM remodeling pathways.
